# Impact of rAAV-shRNA treatment targeting mechanosensitive *Ilk1* and *Fermt2* in a mouse model of sepsis-induced muscle weakness

**DOI:** 10.1371/journal.pone.0338338

**Published:** 2025-12-12

**Authors:** Alexander Pacolet, Inge Derese, Lies Pauwels, Sarah Vander Perre, Maxime Smits, Chris Van den Haute, Sarah Derde, Katrien Koppo, Rik Gijsbers, Lies Langouche

**Affiliations:** 1 Laboratory of Intensive Care Medicine, Department of Cellular and Molecular Medicine, KU Leuven, Leuven, Belgium; 2 ADVANTAGE: Advanced Disease Modeling, Targeted Drug Discovery and Gene Therapy, Department of Pharmaceutical and Pharmacological Sciences, KU Leuven, Leuven, Belgium; 3 Exercise Physiology Research Group, Department of Movement Sciences, KU Leuven, Leuven, Belgium; 4 Research Group for Neurobiology and Gene Therapy, Department of Neuroscience, KU Leuven, Leuven, Belgium; 5 Leuven Viral Vector Core, Biomedical Sciences, KU Leuven, Leuven, Belgium; Zhengzhou University, CHINA

## Abstract

**Introduction:**

Intensive-care-unit-acquired-muscle-weakness is a debilitating complication of sepsis, characterized by loss of muscle mass and functionality. Immobilization is an important trigger, but the role of disturbed mechanical signaling is incompletely understood. In health, the integrin-receptor-complex with key components Kindlin2 (KIND2/*Fermt2*) and integrin-linked-kinase (ILK/*Ilk1*) converses mechanical forces into biochemical signals to regulate muscle mass. We hypothesize that this complex, through key elements KIND2 and ILK, plays a role in sepsis-induced-muscle-weakness.

**Methods:**

AAV2/9-vectors expressing shRNA-sequences against *Ilk1, Fermt2* or noncoding-control-gene were injected in tibialis anterior (TA) muscles of 24w-old male C57BL/6J mice. Two-weeks-post-injection, after knockdown validation, mice were made septic by cecal ligation and puncture. Five-days-post-sepsis muscle force, mass and fiber size were quantified and expression of mechanosensitive elements and downstream pathways of the integrin-receptor-complex was assessed.

**Results:**

Two-weeks-post-injection the respective sh-targets were strongly suppressed (mRNA *Ilk1*–44%, *Fermt2*–76%, protein ILK −34%, KIND2–70%). In rAAV-sh-*controls*, sepsis induced upregulation across TA and EDL muscle of *Ilk1* and *Fermt2* and integrin-receptor-complex-related genes *Itga*7, *ItgB1*, *Tln1*, *Lims1, Lims2, Parva* (*P < 0.001*), whereas in SOL muscle *Lims1, Lims2* and *Fermt2* were not and *Vcl1* (*P < 0.001*) was upregulated. In TA and EDL, but not in SOL, rAAV-sh*Ilk1* and rAAV-sh*Fermt2* attenuated upregulation of respective targets down to healthy controls, but without affecting expression of other integrin-receptor-complex-related genes. TA muscle force or weight were not affected by rAAV-sh*Ilk1* or rAAV-sh*Fermt2* (*P > 0.05*), whereas muscle fiber size reduction (−20.7% in Sepsis shControl) was attenuated up to −13.4% (Sepsis sh*Ilk1*, *P < 0.001*) and −12.3% (Sepsis sh*Fermt2*, *P < 0.001*). Sepsis or sh-treatment did not shift TA fiber types. Expression of markers of atrophy, inflammation, autophagy, protein synthesis and regeneration were affected by sepsis, but not by sh-treatment. Only markers of metabolism *Slc2a4* (*P < 0.05)* and *Rac1* (*P < 0.01*) were further affected by sh-treatment.

**Conclusions:**

Sepsis induced upregulation of integrin-receptor-complex-related genes but attenuating the upregulation of *Ilk1* or *Fermt2* did not affect the development of muscle weakness, although muscle fiber size was better preserved, arguing against a key role for *Ilk1* or *Fermt2*.

## Introduction

The majority of critically ill patients requiring intensive care for more than a few days develop ICU-acquired muscle weakness (ICUAW), characterized by loss of muscle strength and/or muscle atrophy [1]. This complication of critical illness can be prompted by critical illness polyneuropathy, critical illness myopathy, or both and is associated with increased mortality and impaired post-ICU recovery [2]. The pathophysiological mechanisms underlying ICUAW are multifactorial, with sepsis, characterized by elevated levels of inflammatory cytokines and catabolic factors [3], and immobilization identified being the major risk factors [4–6].

Outside the context of critical illness, the effects of immobilization on skeletal muscle integrity and function have been studied primarily using animal models such as hindlimb unloading, denervation, or joint fixation. However, these models produce phenotypes that differ from those observed in critical illness. Indeed, sedated, mechanically ventilated ICU patients experience a state of complete “mechanical silencing”. A rat model replicating these basic ICU conditions has suggested a role for mechanosensing pathways in the development of ICUAW [7]. However, only a subset of critically ill patients undergoes complete sedation, while the majority retain some ability to activate their muscles and process mechanical load. Nevertheless, alterations in mechanical stimuli may still be detected and potentially contribute to ICUAW, as muscle cells possess the intrinsic ability to sense and convert mechanical forces into biochemical events, a process referred to as mechanotransduction [8–10].

The skeletal muscle membrane houses multiple mechanosensitive structures including the integrin receptor complex (IRC). This complex is a large protein assembly where integrins, transmembrane heterodimeric molecules consisting of an α- and a β-subunit, promote mechanical signal transduction in a bi-directional manner [11,12]. Upon activation, integrins adopt a conformation with high affinity for ligands from the extracellular matrix. Integrin activation is a tightly regulated process in which Kindlin2 (KIND2/*Fermt2*), among others, has a critical role [13–15]. KIND2 orchestrates myocyte elongation and myogenesis [16] and directly interacts with the cytoplasmic tail of the integrin β-subunit promoting the link with the cytoplasmic integrin-linked kinase1 (ILK1/*Ilk1*) -pinch-parvin (IPP) complex. This complex forms a signaling platform that links integrins with the actin cytoskeleton and many other signaling pathways essential for skeletal muscle mass regulation [12]. The connection between the extracellular matrix and the cytoskeleton through the interaction of integrins and the IPP, directly linking the nucleus and mitochondria, allows rapid signal propagation to nuclear and mitochondrial DNA, affecting muscle gene expression [17–19]. This hard-wired connection suggests that the mechanosensitive IRC may contribute to both hypertrophic and atrophic processes in skeletal muscle, as indeed observed in previous research [19].

Whether the development of ICUAW, in which sepsis contributes alongside immobilization [7], may be identified as a pathological process influenced by mechanosensitive elements, requires further investigation. We hypothesized that disruption of specific mechanosensing elements would worsen the phenotype of ICUAW. To test this hypothesis, we used a centrally catheterized mouse model of cecal ligation and puncture (CLP)-induced septic critical illness [20–22]. This validated, fluid-resuscitated, antibiotics- and analgesic-treated mouse model is a clinically relevant model of prolonged sepsis, providing intensive care and characterized by the development of ICUAW, with similar features as in humans. Two weeks prior to sepsis induction, the *tibialis anterior* muscles (TA) of the mice were injected with adeno-associated viral vectors (AAV) encoding short-hairpin RNA (shRNA) specifically targeting *Ilk1* or *Fermt2* mRNA. Following five days of sepsis, we assessed the impact of sepsis on muscle integrity and weakness in control, and *Ilk1* or *Fermt2* mRNA depleted conditions. A better insight in the pathophysiology of ICUAW could potentially open therapeutic options to this debilitating condition.

## Materials and methods

### Recombinant adeno-associated viral vector production

Recombinant AAV were produced using a three-plasmid system including the construct for the AAV2/9 serotype that was modified for enhanced intramuscular gene delivery by introducing insulin-mimetic peptide S519 [23]. The AAV transfer plasmids encoded a miR-based shRNA targeting *Ilk1* or *Fermt2* mRNAs in combination with a 3xhemagglutini-tag codon optimized firefly luciferase under the control of the ubiquitous CMV promotor, respectively, resulting in rAAV-sh*Ilk1* and rAAV-sh*Fermt2* viral vector preps. Controls, vectors expressing a miR-based shRNA against Red Fluorescent Protein – not present in wild type mice – were generated (rAAV-shControl).

The rAAV were produced by the Leuven Viral Vector Core as reported earlier [24,25] with minor modifications. Briefly, subconfluent adherent HEK 293T cells (ATCC, Manassas, VA, USA) were transfected using a 25-kDa linear polyethylenimine solution using the AAV-TF, AAV rep/cap and pAdDeltaF6 plasmids in the ratio of 1:1:1. Productions were performed using an 8-layer Celldisc (2000 cm^2^ growth area, Greiner Bio-One). 293T cells were seeded at 350 x 106 cells per production in DMEM (Gibco, Fisher Scientific) with 2% fetal calf serum. The next day, 260 µg of pAAV-TF plasmid, 260 µg of rep/cap construct using the construct for the AAV2/9 serotype that was modified for enhanced intramuscular gene delivery by introducing insulin-mimetic peptide S519 [23] and 260 µg of pAdDeltaF6 plasmid were mixed in 20 ml of PBS. An equal volume containing 8.7 ml of 13 µM polyethylenimine solution and 11.3 ml of PBS was added and following short incubation at room temperature, the complex was added to the 293T cells. After 24 h of incubation at 37°C in a 5% CO_2_ humidified atmosphere, the medium was replaced with Optimem (Gibco, Fisher Scientific) without serum. Medium was harvested for 5 consecutive days and concentrated using tangential flow filtration. The concentrated supernatant was processed using a discontinuous iodixanol step gradient. Iodixanol (60%) was diluted with PBS containing a final concentration of 1 M NaCl in final 20, 30 and 40% (w/v) solutions. The gradient was prepared by carefully underlayering the concentrated supernatant with 5 ml of 20% iodixanol, 3 ml of 30% iodixanol, 3 ml of 40% iodixanol and 3 ml of 60% iodixanol solutions, respectively. Following centrifugation (Beckman Ti-70 fixed angle rotor (Analis, Gent, Belgium) at 27 000 rpm for 2 h), gradient fractions were collected and real-time PCR was performed to identify fractions containing AAV vector using a primer probe set for the BGHpolyA sequence (primer sequences, 5’-TCTAGTTGCCAGCCATCTGTTGT-3’ and 5’-TGGGAGTGGCACCTTCCA-3’; probe sequence, 5’-TCCCCCGTGCCTTCCTTGACC-3’). The pooled positive fractions were centrifuged in a Vivaspin 6 (PES, 100,000 MWCO, Sartorius AG, Goettingen, Germany) using a swinging bucket rotor at 3000 g. The iodixanol of the pooled fractions was replaced by a five-fold exchange with PBS. The final concentrate was aliquoted and stored at −80°C. Final titers of the rAAV stocks were determined by real-time PCR analysis for genomic copy determination and Ruby-stained SDS-PAGE analysis for vector purity.

### Animal study design

In the validation experiment, male 24-week-old C57BL/6J mice (Janvier Labs, Le Genest-Saint-Isle, France) were anaesthetized with isoflurane and injected in the medial aspect of the left tibialis anterior (TA) muscles with 5x10^9^ vector genomes of rAAV-sh*Ilk1* (sh*Ilk1*, n = 4) or rAAV-sh*Fermt2* (sh*Fermt2,* n = 4) in 20 µL PBS, according to test data. The non-injected, right TA muscle served as an intra-animal control and was injected with the rAAV-shControl (shControl). Two weeks post-injection, mice were anaesthetized and sacrificed by cardiac puncture and muscle tissue was harvested to assess gene and protein expression using RT-qPCR and western blot analysis, respectively.

In the sepsis experiment we also used 24-weeks-old mice (mature adult) as this age corresponds better with the mean age of intensive care patients. We used only male mice to avoid the cyclic influence of estrogens. The animals were anaesthetized with isoflurane and after random allocation, injected in the medial aspect of the right and left TA r muscles with rAAV-sh*Ilk1* (Sepsis sh*Ilk1*, n = 23), rAAV-sh*Fermt2* (Sepsis sh*Fermt2*, n = 23) or rAAV-shControl (n = 42). Mice injected with the latter, were randomized to septic controls (Sepsis shControl, n = 22) or a healthy control group (Healthy shControl, n = 20). Transduction efficiency was assessed by measuring luciferase activity using bioluminescence imaging instruments (Perkin-Elmer, Hopkinton, MA, USA) two weeks post-injection. Next, mice in the sepsis groups were anaesthetized with an intraperitoneal injection of ketamine/xylazine (100 mg/kg ketamine, Eurovet Animal Health BV, Bladel, The Netherlands, and 13 mg/kg xylazine, V.M.D. nv/sa, Arendonk, Belgium) and a central venous catheter was implanted in the left jugular vein, after which CLP was applied with a 18-gauge needle to induce sepsis [20]. The chronic indwelling catheter was tightly secured in the vein with silk sutures and tunneled to the back of the animal were it was secured to a metal wire and connected to the rotating point of a swivel, allowing the animal to move freely in its cage. In the first 24 hours after surgery, septic mice received continuous intravenous fluid resuscitation (Plasmalyte, Baxter, Braine-l’Alleud, Belgium, containing 4.2% glucose) at 0.3 ml/h. Hereafter parenteral nutrition (Oliclinomel N7E, Baxter) was administrated at 0.2 ml/h (up to 45% of the normal daily caloric intake, to mimic the illness-induced lack of feeding in human sepsis patients) until the end of the study period. No additional oral chow was provided to keep the nutritional intake equal between septic groups. All septic mice were treated with broad-spectrum antibiotics (Imipenem/Cilastin, Aurobindo Pharma, Hyderabad, India) and opioid-analgesics (Buprenorphine (Vetergesic), Patheon UK Ltd, Covingham, United Kingdom) via subcutaneous injection twice daily. Pain/discomfort was assessed twice daily based on the Mouse Grimace Score and analyzed as a cumulative score across the day study period [26]. Healthy control mice were caged separately and were provided with standard chow and water ad libitum. After 5 days of sepsis, the equivalent of prolonged critical illness in humans, mice were anaesthetized with ketamine/xylazine, *in situ* force measurements were performed (see below) and sacrificed by cardiac puncture. Animals were weighed both at the start and the end of the experiment. To minimize confounders, animals were always operated (mornings) and sacrificed (afternoons) at the same time of day. All animal cages were kept in an animal cabinet under controlled temperature (27°C) and 12 hours light and dark cycles. The primary endpoint was a further decrease in TA muscle mass of critical ill mice by silencing mechanosensors integrin-linked kinase (*Ilk1*) or kindlin-2 (*Fermt2*). Based on an expected effect size of 1.052, a power of 80% and 95% confidence level, we calculated that 16 animals per group were required. To take into account exclusions due to technical problems with the viral injection, the catheter (blocked or dislocated), muscle force measurements (rupture) and the expected 20% mortality of sepsis [20], we randomized 24–26 animals to each group. All animals were treated according to the Principles of Laboratory Animal Care (US National Society of Medical Research) and to the European Union Directive (2010/63/EU) concerning the welfare of laboratory animals and complied with the ARRIVE guidelines [27]. The animal study protocols were registered with and approved by the Institutional Ethical Committee for Animal Experimentation of the KU Leuven (P010-2022, initial approval date of July 1^st^ 2022). All animal experiments were conducted in the Laboratory of Intensive Care Medicine between July 03^th^ 2023 and May 9^th^ 2024. Caretakers and data collectors were blinded for group allocation.

### *In situ* force measurements

In the sepsis experiment, prior to sacrifice, anaesthetized mice were placed on a heating pad and the left TA muscle was exposed followed by detachment and suture of the distal tendon. Then, the animal was placed on the heated (37°C) 809C *in situ* mouse apparatus (Aurora Scientific, Aurora, ON, Canada) where the paw was fixated to the platform as well as the knee joint. The sutured TA tendon was attached to a lever arm (300C-LR Dual-Mode Muscle Lever, Aurora Scientific) which was aligned to match the direction of the TA muscle’s contraction. The TA was kept moist with warm saline. Electrodes were placed on the medial aspect of the TA and resting length was set to 50 mN passive force, as determined by test data. Supramaximal stimulation intensities were determined for each individual muscle by gradually increasing the stimulation intensity until twitch forces plateaued, thereby identifying the optimal muscle length (L_0_). Maximal isometric tetanic forces were measured as the average of three consecutive tetanic stimuli (200 Hz stimulation frequency, 200 ms duration, 0.2 ms pulse width) with 2-minute rest intervals between stimuli. The specific maximal isometric tetanic force was calculated by dividing the muscle mass by the product of the density of mammalian skeletal muscle (1.06 mg/mm^3^) and the optimal fiber length (L_f_ = 0.44 x L_0_) Data analysis was performed with the Dynamic Muscle Analysis Software (Aurora Scientific).

### Gene and protein expression analyses

Immediately after sacrifice, the right TA muscle, soleus muscle (SOL) and extensor digitorum longus muscle (EDL) were carefully dissected, weighed and snap-frozen in liquid nitrogen of all animals surviving until the planned end of the experiment. Total RNA was isolated (RNeasy Mini Kit, Qiagen) and reverse-transcribed into complementary DNA (Superscript lll 1^st^ Strand Synthesis System, Invitrogen). The quantity of the genes of interest in each sample was normalized to that of *Gapdh* using the comparative 2-ΔΔCt method and expressed as fold change of the mean of healthy control samples. A list of all used gene expression assays for markers of mechanosensation, inflammation, fibrosis, protein synthesis, metabolism, mitochondrial regulation and muscle wasting is provided in S1 Table. Protein content in TA muscle of ILK1 (#3862, Polyclonal Rabbit Ab, Cell Signaling, 1/1000) and KIND2 (#13562, Polyclonal Rabbit Ab, Cell Signaling, 1/750) was quantified by western blot analyses. Equal amounts of protein were loaded on a 10% tris-glycine polyacrylamide gel (Bio-rad, Herculus, CA, USA) and separated by SDS-PAGE. Afterwards proteins were electroblotted on a polyvinylidene fluoride (PVDF) membrane which was subsequently blocked with 5% BSA. Overnight incubation at 4°C with primary antibodies and horseradish peroxidase (HRP) linked secondary antibody (Dako, Glostrup, Denmark) at room temperature for 1 h, allowed protein visualization and quantification using chemiluminescence plus technology (PerkinElmer, Zaventem, Belgium) with the G:BOX Chemi XRQ (SynGene, Cambridge, United Kingdom). Images were analyzed with SynGene software.

### Histology

Laminin immunofluorescence staining was conducted on 5 µm-thick paraffin-embedded muscle tissue sections from the left TA to measure the fiber cross-sectional area. Tissue sections were incubated overnight (4°C) with an anti-laminin primary antibody ab11575 (Abcam, Cambridge, UK). Segmentation of the myofibers was performed using Cellpose, and fiber cross-sectional area was measured using the LabelsToROI plugin in ImageJ [28]. For muscle fiber type staining, cryosections of left TA were blocked with mouse-on-mouse blocking reagent and incubated overnight with primary antibodies BA-F8-s, SC-71-s and BF-F3c (Developmental Studies Hybridoma Bank, Iowa, USA). After washing with PBS, cryosections were incubated with conjugated secondary antibodies IgG2b-AF350, IgG1-Cys5 and IgM-AF594 (Thermo Fisher Scientific, Waltham, USA) to identify fiber types l, lla, llx and llb. Images were taken with a TissueFAXS i Plus (TissueGnostics, Vienna, Austria) and analyzed with ImageJ software to determine the fiber type composition.

### Statistical analyses

All data were evaluated for normality using the Shapiro-Wilk test. In the validation experiment, paired-t-tests were used, in the sepsis experiment data were analyzed using a one-way ANOVA followed by post hoc Tukey for multiple comparisons test for normally distributed data and Kruskal-Wallis followed by Dunn’s post hoc test for multiple comparisons for non-normally distributed data. A *P-value* ≤*0.05* was considered statistically significant. Statistical analyses were performed with JMP Pro 17.0.0 (SAS Institute Inc., Carry, NC, USA) and GraphPad Prism 8.4.3 (GraphPad Software, Boston Massachusetts, USA). Data are presented as box plots with median, interquartile ranges and upper and lower adjacent values shown as whiskers, or as bars with whisker, representing the mean and standard error of the mean (SEM). Post hoc values *≤0.05* are indicated on the figures.

## Results

### Validation of the rAAV-sh*Ilk1* and rAAV-sh*Fermt2* on gene and protein expression of their targets

**Fig 1:**
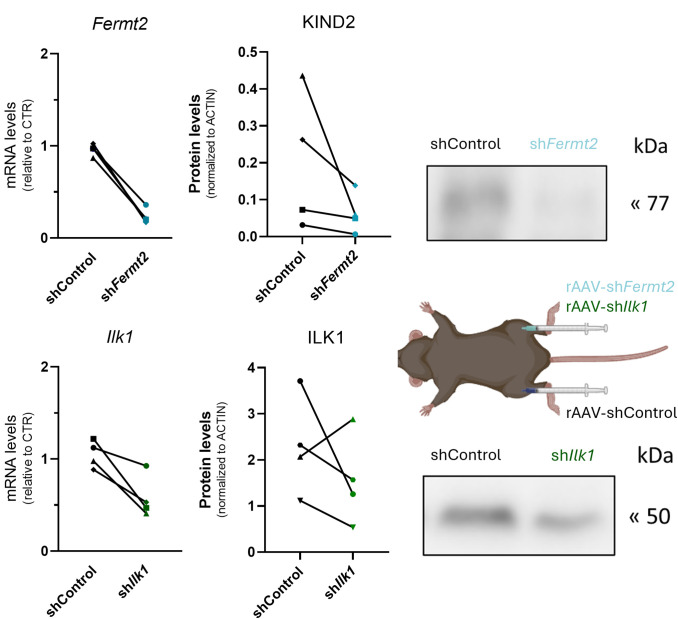
Efficient in vivo knockdown of Fermt2 and Ilk1 in skeletal muscle following intramuscular delivery of AAV2/9-vectors containing targeted shRNA sequences. (**a**) mRNA and protein levels of Ilk1 and protein ILK1 (~50 kDa) compared to contralateral control muscles. Representative western blot and quantification are shown. (**b**) mRNA and protein levels of Fermt2 and its encoded protein KIND2 (~77 kDa) compared to contralateral control muscles. Representative western blot and quantification are shown.

Two weeks post injection, *Fermt2* (Fig 1a) and *Ilk1* (Fig 1b) mRNA levels were reduced with respective 76% and 44% in muscles injected with their respective targeting vectors compared to control-injected contralateral muscles. This efficient knockdown at transcript level was further reflected at the protein level as the expression of KIND2, the protein encoded by *Fermt2* (Fig 1a), and ILK1 (Fig 1b) was decreased with respective 70% and 34% in knockdown samples, as evidenced by western blot analysis and quantification.

### The effect of TA injection of rAAV-sh*Ilk1* or rAAV-sh*Fermt2* on AAV transduction, survival and severity of illness in septic mice

**Fig 2:**
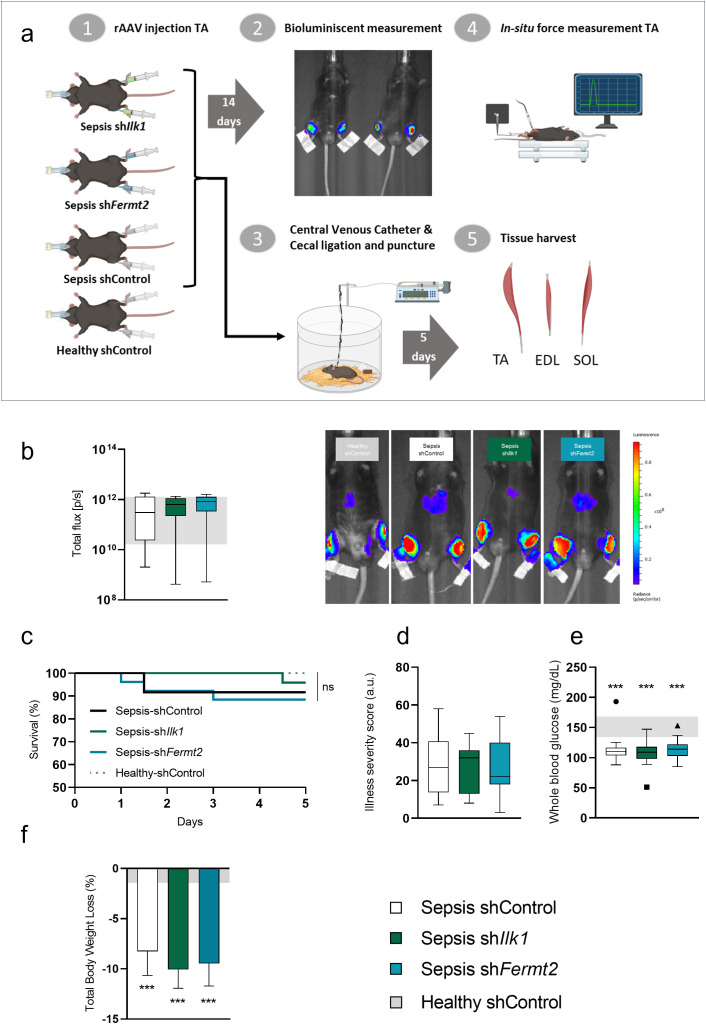
Impact of tibialis anterior injection with rAAV-shIlk1 or rAAV-shFermt2 on AAV transduction, survival and severity of illness in septic mice. **a**: Schematic overview of the experimental setup. **b**: Quantification (total photon flux) and in vivo visualization (bioluminescence images) of luciferase activity two weeks post AAV injection in tibialis anterior muscles of mice. **c**: Survival curves of healthy control mice (n = 20/20), septic rAAV-shIlk1-injected mice (n = 23/24), septic rAAV-shFermt2-injected mice (n = 23/26) and septic control mice (n = 22/24) for the five-day study period. **d**: Cumulative illness severity scores of septic rAAV-shIlk1-injected mice, septic rAAV-shFermt2-injected mice and septic control mice. **e**: Whole blood glucose concentrations on day 5. **f**: Total body weight loss over five days of sepsis. Data presented as box and whiskers plots with median, interquartile ranges and upper and lower adjacent values. Gray area represents the interquartile range of the healthy controls. */**/*** P ≤ 0.05/ P ≤ 0.01/ P ≤ 0.001 compared with healthy control mice.

In the sepsis experiment, when sepsis was induced by cecal ligation and puncture (2 weeks post rAAV injection), muscle tissue was not yet accessible for gene or protein assessment of *Ilk1*/ILK1 and *Fermt2*/KIND2 (Fig 2a). Therefore, we assessed eAAV2/9 transduction *in vivo* by measuring luciferase activity using bioluminescence imaging two weeks post-injection in the TA muscles (Fig 2b). *In vivo* whole-body bioluminescence imaging scans showed photon flux as a result of luciferase expression in the lower limb regions, indicating successful local transduction. Some photon flux was observed in the liver region suggesting minor systemic AAV leakage. Comparison of luciferase activity did not show differences between groups, although variability among animals within groups was observed.

Over five days of sepsis, survival of **r**AAV**-**sh*Ilk1* (96%, 23/24) or rAAV-sh*Fermt2* (89%, 23/26) treated mice was comparable to septic controls (92%, 22/24) (*P = 0.4*) (Fig 2c). Cumulative illness severity scores were similar among septic groups (*P = 0.9*) and elevated compared to controls (Fig 2d). Blood glucose levels were lower compared to healthy control mice (*P < 0.001*), but similar among the 3 septic groups (Fig 2e). Over the course of five days of sepsis and severely reduced physical activity (S1 Fig), all septic mice lost a comparable amount of body weight (~10%, *P < 0.001*) in contrast to healthy control mice (Fig 2f).

### The effect of TA injection of rAAV-sh*Ilk1* or rAAV-sh*Fermt2* on IRC/IPP elements in hindlimb muscles in septic mice

**Fig 3:**
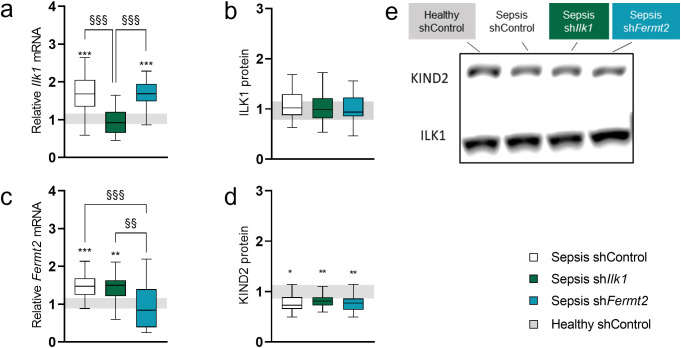
Ilk1/ILK1 and Fermt2/KIND2 gene and protein expression levels in rAAV-injected tibialis anterior muscle. **a**: Relative mRNA expression of Ilk1 in tibialis anterior muscle normalized to Gapdh housekeeping gene and expressed as fold change of the mean of healthy control mice. **b**: Relative protein levels of ILK1 in tibialis anterior muscle. **c**: Relative mRNA expression of Fermt2. **d**: Relative protein levels of KIND2. **e**: Representative images of western blot analysis of ILK1 (~50 kDa) and KIND2 (~77 kDa) protein content. Data presented as box and whisker plots with median, interquartile ranges and upper and lower adjacent values. Gray area represents the interquartile range of the healthy controls. */**/*** P ≤ 0.05/ P ≤ 0.01/ P ≤ 0.001 compared with healthy control mice. §/§§/§§§ P ≤ 0.05/ P ≤ 0.01/ P ≤ 0.001 compared among septic mice.

After five days of sepsis, both *Ilk1* and *Fermt2* mRNA levels were elevated in septic control mice compared to healthy control mice. This upregulation was no longer present in septic mice respectively injected with rAAV-sh*Ilk1* or rAAV-sh*Fermt2*, showing *Ilk1* (*P > 0.9*) or *Fermt2* (*P > 0.9*) expression levels similar to those of healthy control mice (Fig 3a, 3c). After five days of sepsis, ILK1 protein expression in septic mice remained comparable to that of healthy control mice, with no effect of rAAV-sh*Ilk1* or rAAV-sh*Fermt2* (Fig 3b, 3e). The expression of KIND2, encoded by *Fermt2*, was reduced in all septic mice compared to healthy controls, with no significant differences among groups (Fig 3d, 3e).

**Fig 4:**
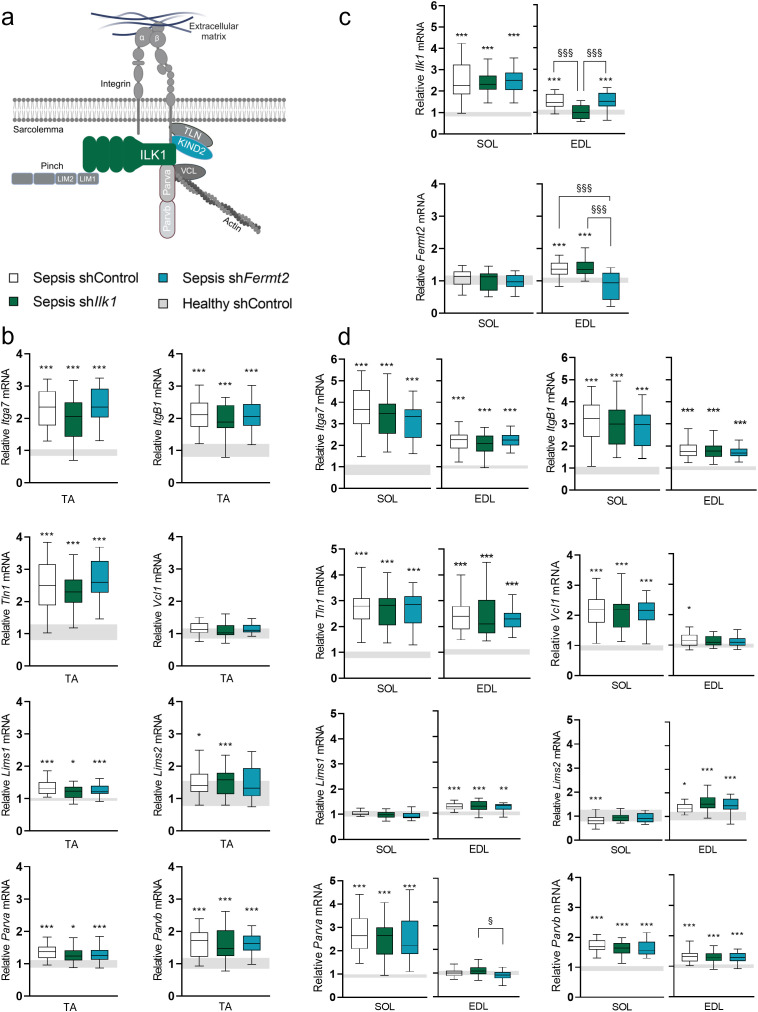
Gene expression of IRC/IPP elements in rAAV-injected tibialis anterior muscle, in soleus muscle and in extensor digitorum longus muscle. a: Schematic representation of the IRC/IPP complex (Created with Biorender.com). b: Relative mRNA expression of IRC/IPP – related genes (Itga7, Itgb1, Tln1, Vcl1, Lims1, Lims2, Parva, Parvb) in tibialis anterior muscle. c: Relative mRNA expression of Ilk1 and Fermt2 in soleus and extensor digitorum longus muscle. d: Relative mRNA expression of IRC/IPP – related genes (Itga7, Itgb1, Tln1, Vcl1, Lims1, Lims2, Parva, Parvb) in soleus and extensor digitorum longus muscle. Data is normalized to Gapdh housekeeping gene and expressed as fold change of the mean of healthy control mice. Data presented as box and whisker plots with median, interquartile ranges and upper and lower adjacent values. Gray area represents the interquartile range of the healthy controls. */**/*** P ≤ 0.05/ P ≤ 0.01/ P ≤ 0.001 compared with healthy control mice. §/§§/§§§ P ≤ 0.05/ P ≤ 0.01/ P ≤ 0.001 compared among septic mice.

Next, we assessed key components of the integrin-receptor complex (Fig 4a) in TA. In line with *Ilk1* and *Fermt2*, genes encoding for the integrin subunits (*Itga7* and *ItgB1*) were upregulated (more than 2-fold; *P < 0.001* versus healthy control mice) after five days of sepsis (Fig 4b). Also, the expression of *Tln1,* encoding a protein involved in integrin activation was increased (*P < 0.001* versus healthy control mice), whereas *Vcl1* mRNA levels remained unchanged (Fig 4b). Similarly, genes encoding structural components of the IPP assembly including *Lims1* (Pinch-1) and *Lims2* (Pinch-2), *Parva* (α-Parvin) and *Parvb* (β-Parvin) were upregulated (Fig 4b). Gene expression of none of these IRC/IPP elements was affected by the attenuated upregulation of *Ilk1* or *Fermt2,* with no significant differences between the septic groups.

As previous research suggested mechanosensitive gene expression to be muscle fiber type-specific explaining adaptation differences to mechanical loading [29], mRNA expression levels of IRC/IPP elements was analyzed in SOL muscle, which is predominantly composed of oxidative fibers. In SOL muscle, gene expression of *Ilk1* was upregulated compared to healthy control mice (*P < 0.001*, Fig 4c), whereas the expression of *Fermt2* was not affected by sepsis (*P = 0.6*, Fig 4c). Conversely, while no alteration in *Vcl1* expression was detected in TA muscle of septic mice, this gene, encoding for an accessory molecule of the IRC/IPP complex, was upregulated in SOL muscle (Fig 4d). Again, in contrast to TA, expression of *Lims1* and *Lims2* was not upregulated in SOL muscle (Fig 4d). On the other hand, the expression of *Itga7*, *ItgB1*, *Tln1*, *Parva* and *Parvb* were all similarly upregulated in SOL in septic mice compared to healthy controls (Fig 4d).

The EDL muscle is predominantly composed of fast-twitch type ll muscle fibers [30] but is also located close to the TA muscle, so a spill-over effect of rAAV-injection can be expected. Indeed, where *Ilk1* and *Fermt2* gene expression was similarly upregulated as in the TA in septic control mice, this was prevented in septic mice injected with rAAV-sh*Ilk1* or rAAV-sh*Fermt2*, respectively (Fig 4c). Similar to the TA, gene expression of *Itga7*, *ItgB1*, *Tln1*, *Vcl1* and *Parvb* was upregulated in sepsis (Fig 4d). Nevertheless, also here the attenuated upregulation of *Ilk1* or *Fermt2* did not interfere with expression of the other IRC/IPP related genes, except for *Parva*, for which the rAAV-sh*Ilk1* group demonstrated a slightly higher expression level compared to the rAAV-sh*Fermt2* treated group (*P = 0.03*, Fig 4d).

### The effect of TA injection of rAAV-sh*Ilk1* or rAAV-sh*Fermt2* on TA muscle force and muscle mass in septic mice

**Fig 5:**
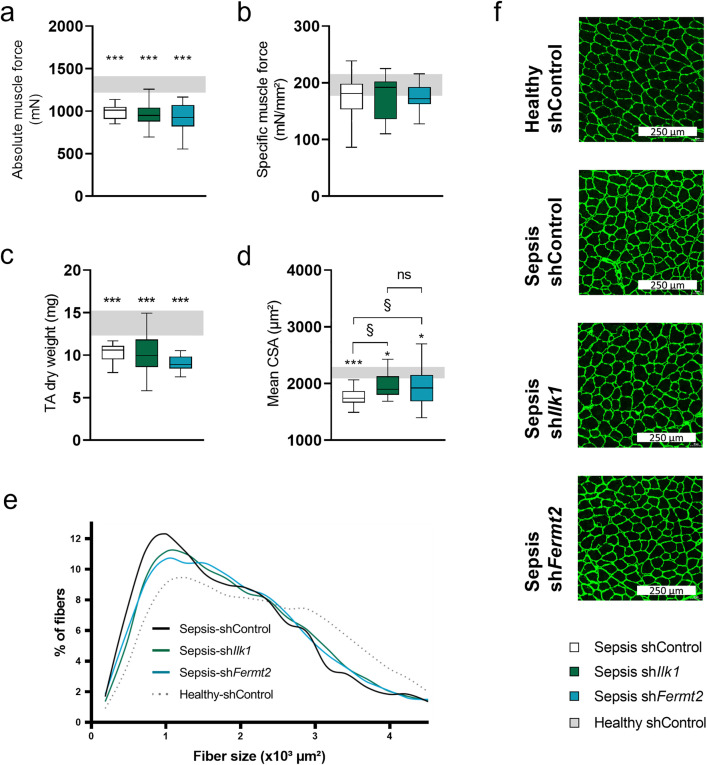
The effect of TA injection with rAAV-shIlk1 or rAAV-shFermt2 on muscle force and muscle mass in septic mice. a: Tibialis anterior absolute muscle force as determined by in situ measurement. b: Tibialis anterior muscle force corrected for whole muscle cross-sectional area. c: Tibialis anterior muscle dry weight. d: Average fiber size for all fibers (µm^2^). e: frequency histograms showing the distribution of cross-sectional areas (103 x µm^2^) f: Tibialis anterior sections stained for laminin. Data presented as box and whisker plots with median, interquartile ranges and upper and lower adjacent values. Gray area represents the interquartile range of the healthy controls. */**/*** P ≤ 0.05/ P ≤ 0.01/ P ≤ 0.001 compared with healthy control mice. §/§§/§§§ P ≤ 0.05/ P ≤ 0.01/ P ≤ 0.001 compared among septic mice.

To assess muscle functionality upon ICU-conditions in absence of *Ilk1* or *Fermt2* gene upregulation, *in situ* force measurements were performed on the rAAV-injected left TA muscle. Absolute muscle force generation was significantly lower (~30%, *P < 0.0001*) in all septic mice, irrespective of rAAV treatment, compared to healthy mice (Fig 5a). Specific muscle force, which is controlled for the cross sectional-area of the TA, did not differ between septic and healthy mice (Fig 5b). Neither absolute or specific muscle force was affected by the rAAV-sh*Ilk1* or rAAV-sh*Fermt2* injection.

To assess skeletal muscle wasting after five days of sepsis, TA dry weight was measured to eliminate variability caused by edema. Total TA muscle dry weight was decreased by ~30% (*P < 0.001*, Fig 5c) in all septic groups compared to healthy control mice, not affected by *Ilk1* or *Fermt2* knockdown. Also mean cross-sectional area of individual muscle fibers was decreased in control septic mice compared to healthy control mice**.** Remarkably, muscle cells with attenuated *Ilk1* or *Fermt2* upregulation demonstrated to suffer less from cross-sectional fiber size reduction in comparison with those of septic control mice (*P < 0.05* Fig 5d-5f).

### The effect of TA injection of rAAV-sh*Ilk1* or rAAV-sh*Fermt2* on fiber type, and markers of muscle atrophy, protein synthesis and regeneration in septic mice

**Fig 6:**
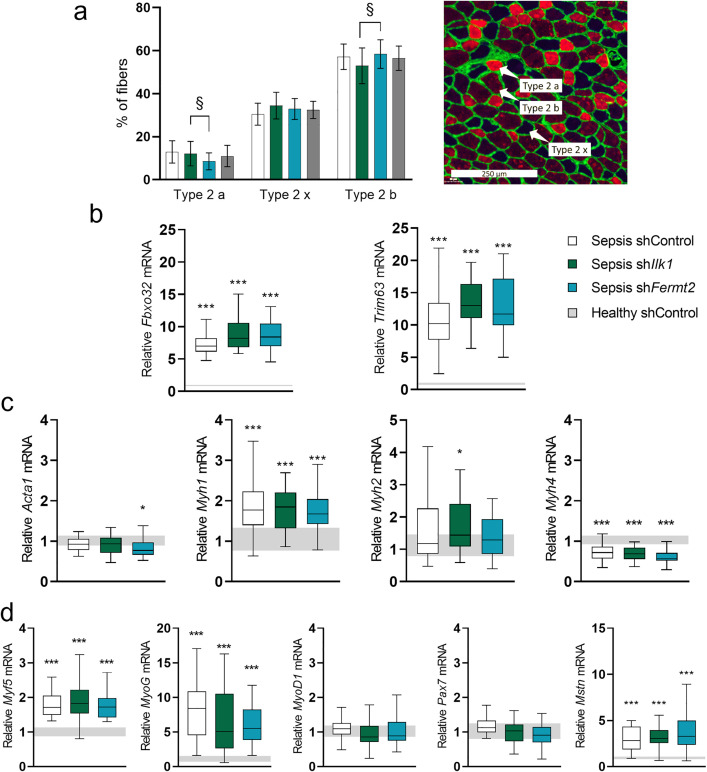
The effect of TA injection with rAAV-shIlk1 or rAAV-shFermt2 on fiber type, and markers of protein synthesis and regeneration in septic mice. a: Percentage of fibers expressing myosin heavy chain types lla, llb and llx proteins (left) and tibialis anterior sections stained for muscle fiber types (right). b: Relative expression of markers of atrophy (Fbxo32 and Trim63) in tibialis anterior muscle. c: Relative expression of markers of protein synthesis (Acta1, Myh1, Myh2, Myh4) in tibialis anterior muscle. d: Relative expression of markers of regeneration (Myf5, MyoG, MyoD1, Pax7, Mstn) in tibialis anterior muscle. Data presented as box and whisker plots with median, interquartile ranges and upper and lower adjacent values. Gray area represents the interquartile range of the healthy controls. */**/*** P ≤ 0.05/ P ≤ 0.01/ P ≤ 0.001 compared with healthy control mice. §/§§/§§§ P ≤ 0.05/ P ≤ 0.01/ P ≤ 0.001 compared among septic mice.

We first investigated whether a shift in fiber type, and associated properties, could explain the improved fiber size preservation observed in rAAV-sh*Ilk1* and rAAV-sh*Fermt2* mice. Immunohistochemical analyses demonstrated that the composition of muscle fiber types was largely unaltered upon sepsis. Only a minor shift from type IIa to llb muscle fibers (*P < 0.05*) was detected in sections comparing rAAV-sh*Ilk1* and rAAV-sh*Fermt2* treated TA (Fig 6a).

Muscle gene expression of atrophy markers *Fbxo32* and *Trim63* were similarly upregulated among the septic groups and were not affected by locoregional rAAV treatment (**Fig 6b**). Since muscle mass is determined by protein breakdown as well as protein synthesis, gene expression of the contractile proteins actin and myosin was quantified in TA muscle. Gene expression of *Acta1* was unaltered upon sepsis, and only rAAV-sh*Fermt2* treatment resulted in a minor decrease (*P < 0.05*, Fig 6c). Sepsis upregulated the expression of *Myh1* in all groups (P < 0.001, Fig 6c), whereas only rAAV-sh*Ilk1* mice displayed increased *Myh2* mRNA expression (*P < 0.05*, Fig 6c). Gene expression of *Myh4* was decreased in all septic groups (*P < 0.001*, Fig 6c).

A shift in fiber size can potentially also be attributed to a shift in regenerative capacity. In TA, sepsis increased expression of markers of muscle regeneration *Myf5* and *Myog*, but not *Pax7* and *Myod1* (Fig 6d). The expression of *Mstn*, a negative regulator of muscle growth, was upregulated by sepsis (W Fig 6d). None of these quantified genes were affected by rAAV-treatment.

### The effect of TA injection of rAAV-sh*Ilk1* or rAAV-sh*Fermt2* on markers of inflammation, fibrosis and autophagy in septic mice

**Fig 7:**
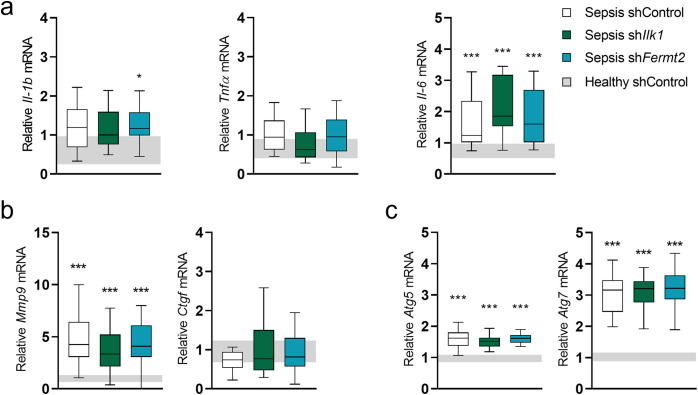
The effect of TA injection with rAAV-shIlk1 or rAAV-shFermt2 on markers of inflammation, fibrosis and autophagy in septic mice. **a**: Relative expression of markers of inflammation (Il-1B, Tnf α and Il-6) in tibialis anterior muscle. **b**: Relative expression of markers of fibrosis (Mmp9 and Ctgf) in tibialis anterior muscle. **c**: Relative expression of markers of autophagy (Atg5 and Atg7) in tibialis anterior muscle. Data presented as box and whisker plots with median, interquartile ranges and upper and lower adjacent values. Gray area represents the interquartile range of the healthy controls. */**/*** P ≤ 0.05/ P ≤ 0.01/ P ≤ 0.001 compared with healthy control mice. §/§§/§§§ P ≤ 0.05/ P ≤ 0.01/ P ≤ 0.001 compared among septic mice.

Inflammation, fibrosis and autophagy are 3 important pathways known to be affected in sepsis, but without a described link with mechanosensing pathways. Gene expression of inflammatory markers *Il-1β* and *Tnfα* remained unaltered compared to healthy controls, whereas *Il-6* expression was increased (*P < 0.001*), but not further affected by rAAV-sh*Ilk1* or rAAV-sh*Fermt2* (Fig 7a). Marker of fibrosis *Mmp9* was increased upon sepsis (*P < 0.001*), but not *Ctgf*, and neither were further affected by rAAV-sh treatment (Fig 7b). Markers of autophagy, *Atg5* and *Atg7*, were upregulated in all septic mice compared to healthy controls, again not affected by rAAV-sh*Ilk1* or rAAV-sh*Fermt2* (*P < 0.001*, Fig 7c).

### The effect of TA injection of rAAV-sh*Ilk1* or rAAV-sh*Fermt2* on markers of metabolism in septic mice

**Fig 8:**
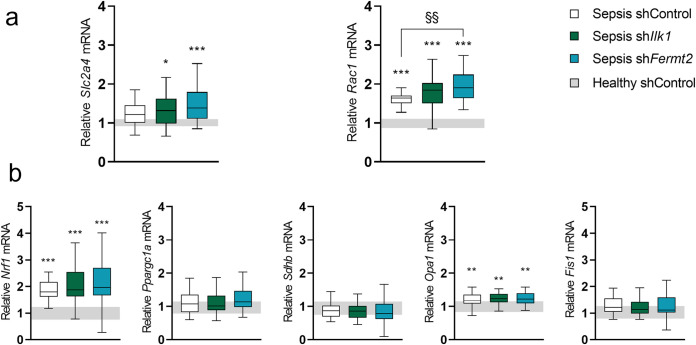
The effect of TA injection with rAAV-shIlk1 or rAAV-shFermt2 on markers of metabolism in septic mice. a: Relative expression of genes involved in glucose transport (Slc2a4 and Rac1) in tibialis anterior muscle. b: Relative expression of genes involved in mitochondrial function (Nrf1, Ppargc1a, Sdhb, Opa1 and Fis1) in tibialis anterior muscle. Data presented as box and whisker plots with median, interquartile ranges and upper and lower adjacent values. Gray area represents the interquartile range of the healthy controls. */**/*** P ≤ 0.05/ P ≤ 0.01/ P ≤ 0.001 compared with healthy control mice. §/§§/§§§ P ≤ 0.05/ P ≤ 0.01/ P ≤ 0.001 compared among septic mice.

As mechanosensors have been shown to affect metabolic markers [31] and as even subtle switch in metabolic profiles could also induce a shift in fiber type and size, we evaluated key markers of muscle metabolism. Gene expression of *Slc2a4*, encoding glucose transporter 4, was upregulated in Septic sh*Ilk1* and Septic sh*Fermt2* mice (*P < 0.05* and *P < 0.001*, respectively) compared to healthy controls but not in Septic shControl mice (*P = 0.2*, Fig 8a). *Rac1,* a gene associated with GLUT4 translocation and related to integrin signaling [32], was upregulated with sepsis (*P < 0.001*), and even further in Septic sh*Fermt2* mice compared to Septic shControl mice (*P < 0.01*, **Fig 8a**). Sepsis significantly increased the expression of *Nrf1* (*P < 0.001,* Fig 8b), a transcription factor involved in the regulation of key metabolic processes, including mitochondrial respiration and DNA transcription and replication. However, mRNA levels of *Ppargc1a*, the master regulator of mitochondrial biogenesis and function, and *Sdhb*, crucial component of the succinate dehydrogenase complex, remained unaltered after five days of sepsis (Fig 8b). *Opa1* expression, contributing to mitochondrial fusion processes, was upregulated with sepsis (*P < 0.01* vs. healthy control mice, Fig 8b), whereas the expression of *Fis1* remained unaltered (*P = 0.2*, Fig 8b). Neither *Nrf1, Ppargc1a, Sdhb*, *Opa1* or *Fis1* were affected by rAAV-sh treatment (Fig 8b).

## Discussion

In this study we first demonstrated strong suppression of gene and protein expression of target genes *Ilk1* and *Fermt2* in TA by locoregional administration of recombinant AAV-vectors containing gene specific shRNA sequences. Next, we demonstrated that sepsis induces the upregulation of key components of the IRC/IPP complex in the hindlimb muscles of mice. Specific rAAV-shRNA treatment in the TA muscle two weeks prior to sepsis induction, attenuated the upregulation of *Ilk1* and *Fermt2* mRNA, but did not affect muscle wasting, weakness or downstream signaling pathways. However, muscle fiber size was better preserved in rAAV-sh*Ilk1*- and rAAV-sh*Fermt2-*treated mice. Notably, sepsis did not induce fiber type shifting in the tibialis anterior muscle, but affected mRNA expression levels of key markers of atrophy, inflammation, autophagy, protein synthesis, and regeneration. Even though AAV injection in TA significantly downregulated the mRNA expression levels for *Ilk1* and *Fermt2* respectively, the effects remained unaltered. Only metabolic markers *Slc2a4* and *Rac1* mRNA levels showed additional changes following rAAV-shRNA treatment.

The combination of cecal ligation and puncture to induce sepsis with the use of a central venous catheter creates a clinically relevant mouse model of critical illness, replicating both the systemic effect of sepsis, with its inflammation and hypercatabolic context [3], and the partial loss of mechanical stimuli [20]. Indeed, although mice are not completely “mechanically silenced” or immobilized, our mouse model of pronged sepsis causes a more than 3-fold reduction in moved distance, as compared to healthy mice. Since decreased muscle use is considered a key factor in the development of ICUAW, understanding the role of mechanosensitive elements, their regulation, and how mechanosensitive pathways respond under these septic conditions is crucial.

In this study we report that five days of sepsis resulted in altered mechanical signaling including changes in IRC/IPP-related gene expression in hindlimb muscles TA, EDL and SOL. While many of these genes have been studied in various models of muscle unloading, our findings present interesting contrasts. Unlike previous reports in adult patients after 5 days of bedrest [33], we observed an upregulation of integrin signaling in our mouse model (*Itga7*, *Itgb1*). Interestingly, *Itga7* and *Itgb1* mRNA levels have also been reported to increase in response to exercise in humans and mice where their upregulation correlates with membrane repair and protection against myofiber disruption [34–36]. Integrins serve as key mediators of bidirectional signaling between the extracellular matrix and the cytoskeleton but rely on KIND2/*Fermt2* and ILK/*Ilk1* for activation and function [37]. ILK, which binds to the cytoplasmic tail of integrin β1, functions as a scaffold protein recruiting IPP-related components such as Pinch, Parvin and Kindlin2. This interaction plays a crucial role in organizing the actin cytoskeleton, thereby coupling mechanical strain to Akt-phosphorylation [12,38], essential for muscle homeostasis and regeneration. It has been reported that ILK-deficient mice develop a mild muscular dystrophy and exhibit increased susceptibility to stress-induced damage [38]. Studies applying hindlimb suspension reported decreased expression levels of *Ilk1* in the unloaded muscles [31], whereas prolonged muscle loading resulted in elevated levels [35]. In contrast, but similar to what was seen in immobilized TA muscles of rats [39], we observed upregulated *Ilk1* expression in TA, SOL and EDL upon sepsis. KIND2 has been identified as an important co-activator of integrins as in KIND2 deficient mice impaired integrin activation led to severe endoderm and epiblast detachments [14,40]. Its role in muscle however, where Kindlin2 is preferentially expressed [41], remains elusive. Studies in cell culture models found that Kindlin2 is crucial for myocyte elongation and myogenesis [16]. Our data shows an upregulation of *Fermt2* gene expression after five days of sepsis in TA and EDL, but not in SOL. These variational patterns are plausibly explained by the differences in muscle fiber type distribution between TA or EDL (predominantly fast-twitch type 2 fibers) and SOL (predominantly slow-twitch type 1 fibers) since these mechanosensitive genes show specific type 1 or type 2 fiber expressions [29]. Likewise, in SOL we found upregulated expression of *Vcl1* and *Parva* which were not detected in EDL muscle.

In TA, the observed upregulation of *Ilk1* and *Fermt2* in TA was successfully prevented in mice treated with rAAV-delivered shRNAs specifically targeting *Ilk1* or *Fermt2* mRNA over the 5 days of sepsis. We realize shRNA treatment only resulted in a normalization of *Ilk1* and *Fermt2* expression and did not achieve higher knockdown rates despite successful transduction with AAV vectors optimized for enhanced intramuscular gene delivery [23]. Also, after 5 days of sepsis, protein levels of ILK1 and KIND2 remained unchanged across groups, and were no longer suppressed by the rAAV-shRNA treatment. This limitation may be attributed to the restricted availability of the RISC complex combined with the sepsis-induced upregulation of the genes of interest, which was observed in this case, ultimately reducing knockdown efficiency. Besides, integrin-linked kinase mRNA and/or protein exhibit a long half-life time in skeletal muscle fibers [38]. More potent knockdown, or the use of a knockout approach could shed a different light on the matter in future studies by using AAV vector that encode CRISPR machinery [42,43]. Of note, in EDL we observed similar expression patterns of *Ilk1* and *Fermt2* compared to the injected TA, suggesting viral vector spill over, explained by its location in close proximity to the TA. The attenuated upregulation of *Ilk1* or *Fermt2* did not lead to differently expressed IRC/IPP-related genes, which is in line with previous research concerning the effect of muscle-specific deletion of *Ilk1* on integrin α7β1 expression [44].

The absolute reduction in muscle force, a hallmark in sepsis primarily driven by altered myofiber structural integrity [22], was not exacerbated by rAAV-sh*Ilk1* or rAAV-sh*Fermt2* treatment. Since Ilk serves as the central hub of the IRC/IPP complex, its downregulation would be expected to impair force transmission and compromise muscle strength. However, over the 5 days of sepsis, rAAV-sh*Ilk1* treatment resulted only in the normalization of *Ilk* gene expression with no longer detectable changes in ILK protein levels. Additionally, the potential loss of ILK and any associated contractile dysfunction may be compensated by other actin-linked adhesion complexes, such as the dystrophin-glycoprotein complex [45,46]. Similarly, the attenuated upregulation of *Fermt2* did not further impair muscle force generation. This contrasts with earlier research where depletion of Kindlin2 has been shown to induce cardiac dysfunction in mice, by a dysregulation of z-disc integrity in cardiac muscles [47].

Remarkably, muscle cells with attenuated *Ilk1* or *Fermt2* mRNA upregulation exhibited better-preserved fiber cross-sectional area compared to septic control mice. However, we did not detect any differences among septic mice regarding TA muscle dry weight. Also, markers of atrophy (*Fbxo32*, *Trim63*) were not differently upregulated. The role of integrins and their associated components in atrophic processes remains underexplored. However, Ilk’s ability to connect the IRC with the insulin-like growth factor I receptor (IGF-IR), thereby coupling mechanical strain to Akt phosphorylation [12,38,48], gives rise to the hypothesis that Ilk has a crucial role in transducing trophic signals. Concerning the role of Kindlin2 in muscle atrophying conditions, not much is known. However, a recent study found that Kindlin2 negatively regulates hypertrophic transcription factors in neonatal mouse cardiomyocytes [49].

We investigated whether the better-preserved fiber cross-sectional area in Sepsis sh*Ilk1* and Sepsis sh*Fermt2* is the result of a potential alteration in fiber type composition. In sepsis, mostly type llb fibers are susceptible for atrophic processes [50,51] and in many cases a shift from fast- to slow-twitch fiber types has been observed [52]. In addition, the aforementioned study suggested that *Ilk* downregulation affects MyHC gene expression towards a phenotype present in slow fibers [29]. Our data do not point toward a shift in muscle fiber type composition, however muscles treated with rAAV-sh*Ilk1* exhibited a lower number of type llb cells compared to rAAV-sh*Fermt2* treated TA muscles. Conversely, this difference was reversed for type lla fibers.

Pathways of inflammation, atrophy, protein synthesis or muscle regeneration are all known to be involved in critical illness induced muscle wasting and weakness [4]. None of these pathways however appeared affected by rAAV-sh*Fermt2* or rAAV-sh*Ilk1*, compared to Sepsis shControl, and could explain the observed attenuated fiber size with both knock downs. We therefore also analyzed markers of metabolism, which were previously demonstrated to be also regulated by mechanosensors [31,53,54]. Although we did not observe a shift in fiber type in TA, more subtle improvements in metabolism or mitochondrial function might potentially also affect the overall size of muscle fibers [55]. We indeed did observe a further increased expression of the GLUT4 receptor and its regulator Rac1, whereas mitochondrial markers were not affected. Whether this pathway truly is involved in the observed change in myofiber size, or is an accidental finding, requires further investigation.

Although our mouse model includes known muscle weakness risks such as prolonged illness, sepsis-induced inflammation and reduced mobility, some limitations need to be addressed. First, we did not include sham-operated mice nor administer antibiotics or pain medication to healthy mice, as we wanted to study the complete phenotype of critical illness caused by the complex interplay of disease and ICU treatments. Furthermore, the complex pathophysiology of muscle weakness in critical illness involves several changes that do not all occur simultaneously. Although the 5-day model of sepsis mimics several aspects of the muscle weakness, some factors may require a more prolonged model combined with complete immobilization and mechanical ventilation. Finally, although the animal model mimics several important characteristics of human critical illness, caution is warranted with extrapolation to human patients. Nonetheless, the use of a clinically relevant sepsis model, optimized AAV vectors for intramuscular delivery, and comprehensive phenotyping—including molecular, histological, and functional assessments—provides robust insight into the role of mechanosensors in sepsis-induced muscle pathology.

In conclusion, sepsis upregulated integrin-receptor-complex-related genes across different hind limb muscles. Attenuating the upregulation of *Ilk1* or *Fermt2* specifically did not affect the development of muscle wasting or weakness, nor its downstream pathways, although muscle fiber size was better preserved. This argues against a key role for *Ilk1* or *Fermt2* in the development of sepsis-induced muscle wasting. The use of the AAV-muscle targeted approach will help further research to identify other potential novel therapeutic strategies to tackle muscle weakness in critically ill patients.

## Supporting information

S1 Table List of used commercially available gene expression assays. (PDF)

S1 Fig Recorded moved distance of healthy controls and septic mice. A noninterfering camera system was used to measure in-cage spontaneous physical activity in 4 healthy controls and 6 septic mice over 3 days (72H) [56]. In 4 healthy control mice and 6 septic mice, spontaneous movement was monitored with a camera tracking system over the course of 3 days. Mice were part of a study published earlier were details on the mouse model and treatment can be found [57]. All septic mice were part of the placebo group. Mice were monitored from the start of sepsis up to 72h later. Following acquisition, video ﬁles were processed and collected data was converted to distance in km [56]. The mean distance moved over 3 days by the healthy mice was 30.6 km (10.2 km per 24h), while this was more then 3-fold reduced in septic mice to 8.8 km (2.9 km per 24h).
(PDF)

S1 File Uncropped and unadjusted images of Western blots. (PDF)

## Abbreviations

AAV: adeno-associated viral vector
ANOVA: analysis of variance
CLP: cecal ligation and puncture
CMV: cytomegalovirus
EDL: extensor digitorum longus muscle
ICU: intensive care unit
ICUAW: intensive care unit- acquired muscle weakness
IPP: integrin-linked kinase-pinch-parvin
IRC: integrin receptor complex
SEM: standard error of mean
sh: short hairpin
SOL: soleus muscle
TA: tibialis anterior muscle
