# Supplementary material for: Impact of rAAV-shRNA treatment targeting mechanosensitive Ilk1 and Fermt2 in a mouse model of sepsis-induced muscle weakness
Source: PLoS One. 2025 Dec 12;20(12):e0338338. doi: 10.1371/journal.pone.0338338 (PMC12700450; doi:10.1371/journal.pone.0338338)
Supplement: S1 Table — (PDF) [file pone.0338338.s001.pdf]

Supplemental table 1: List of used commercially available gene expression assays (Applied Biosystems)

Acta1	Mm008008218_g1
Atg5	Mm01187303_m1
Atg7	Mm00512209_m1
Ctgf	Mm01192933_g1
Fbxo32	Mm00499523_m1
Fermt2	Mm00600590_m1
Fis1	Mm00481580_m1
Fis1	Mm00481580_m1
Il-1β	Mm00443258_m1
Il-6	Mm00446190_m1
Ilk1	Mm01274281_g1
Itgb1	Mm01253230
Lims1	Mm00499506_m1
Lims2	Mm00523019_m1
Mmp9	Mm00442991_m1
Mstn	Mm01254559_m1
Myf5	Mm00435125_m1
Myh1 (IIx)	Mm01332489_m1
Myh2 (IIa)	Mm01332564_m1
Myh4 (IIb)	Mm01332516_g1
Myod1	Mm00440387_m1
Myog	Mm00446194_m1
Nrf1	Mm01135606_m1
Opa1	Mm01349707_g1
Parva	Mm00480444_m1
Parvb	Mm00459990_m1
Pax7	Mm01354484_m1
Ppargc1a	Mm01208835_m1
Rac1	Mm01201653_mH
Sdhb	Mm00458272_m1
Slc2a4	Mm00436615_m1
Tln1	Mm00456997_m1
Tnfα	Mm00443258_m1
Trim63	Mm01185221_m1
Vcl1	Mm00447745_m1
