# Supplementary material for: Impact of rAAV-shRNA treatment targeting mechanosensitive Ilk1 and Fermt2 in a mouse model of sepsis-induced muscle weakness
Source: PLoS One. 2025 Dec 12;20(12):e0338338. doi: 10.1371/journal.pone.0338338 (PMC12700450; doi:10.1371/journal.pone.0338338)
Supplement: S1 Fig — A noninterfering camera system was used to measure in-cage spontaneous physical activity in 4 healthy controls and 6 septic mice over 3 days (72H) [56]. In 4 healthy control mice and 6 septic mice, spontaneous movement was monitored with a camera tracking system over the course of 3 days. Mice were part of a study published earlier were details on the mouse model and treatment can be found [57]. All septic mice were part of the placebo group. Mice were monitored from the start of sepsis up to 72h later. Following acquisition, video ﬁles were processed and collected data was converted to distance in km [56]. The mean distance moved over 3 days by the healthy mice was 30.6 km (10.2 km per 24h), while this was more then 3-fold reduced in septic mice to 8.8 km (2.9 km per 24h). (PDF) [file pone.0338338.s002.pdf]

Supplemental Figure 1

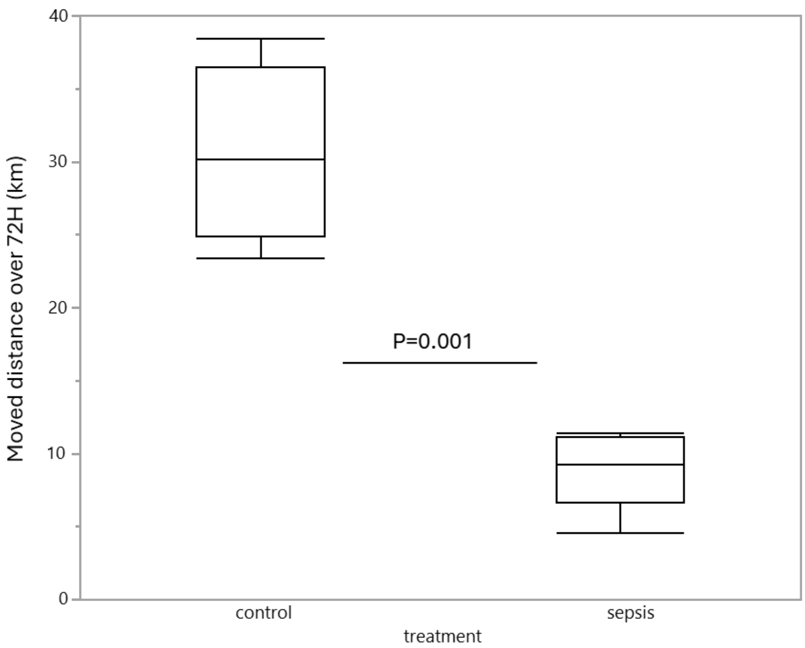

Supplemental Figure 1: **Recorded moved distance of healthy controls and septic mice.** A noninterfering camera system was used to measure in-cage spontaneous physical activity in 4 healthy controls and 6 septic mice over 3 days (72H) (Poffé et al., 2018). In 4 healthy control mice and 6 septic mice, spontaneous movement was monitored with a camera tracking system over the course of 3 days. Mice were part of a study published earlier were details on the mouse model and treatment can be found (Weckx et al, 2021). All septic mice were part of the placebo group. Mice were monitored from the start of sepsis up to 72h later. Following acquisition, video files were processed and collected data was converted to distance in km (Poffé et al., 2018). The mean distance moved over 3 days by the healthy mice was 30.6 km (10.2 km per 24h), while this was more then 3-fold reduced in septic mice to 8.8 km (2.9 km per 24h).

Poffé, C., Dalle, S., Kainz, H., Berardi, E., Hespel, P. (2018). A noninterfering system to measure in-cage spontaneous physical activityin mice. *J Appl Physiol*, *125,* 263-270. https://doi:10.1152/japplphysiol.00058.2018

Weckx, R., Goossens, C, Derde, S, Pauwels, L, Vander Perre, S, Van den Berghe, G., Langouche, L. (2021). Identification of the toxic threshold of 3-hydroxybutyrate-sodium supplementation in septic mice. *BMC Pharmacol Toxicol*, 1:50. https://doi: 10.1186/s40360-021-00517-7.
