# Supplementary material for: Impact of rAAV-shRNA treatment targeting mechanosensitive Ilk1 and Fermt2 in a mouse model of sepsis-induced muscle weakness
Source: PLoS One. 2025 Dec 12;20(12):e0338338. doi: 10.1371/journal.pone.0338338 (PMC12700450; doi:10.1371/journal.pone.0338338)
Supplement: S1 File — (PDF) [file pone.0338338.s003.pdf]

**Supplemental data 1** – Raw Images Western Blots

**FIGURE 1**

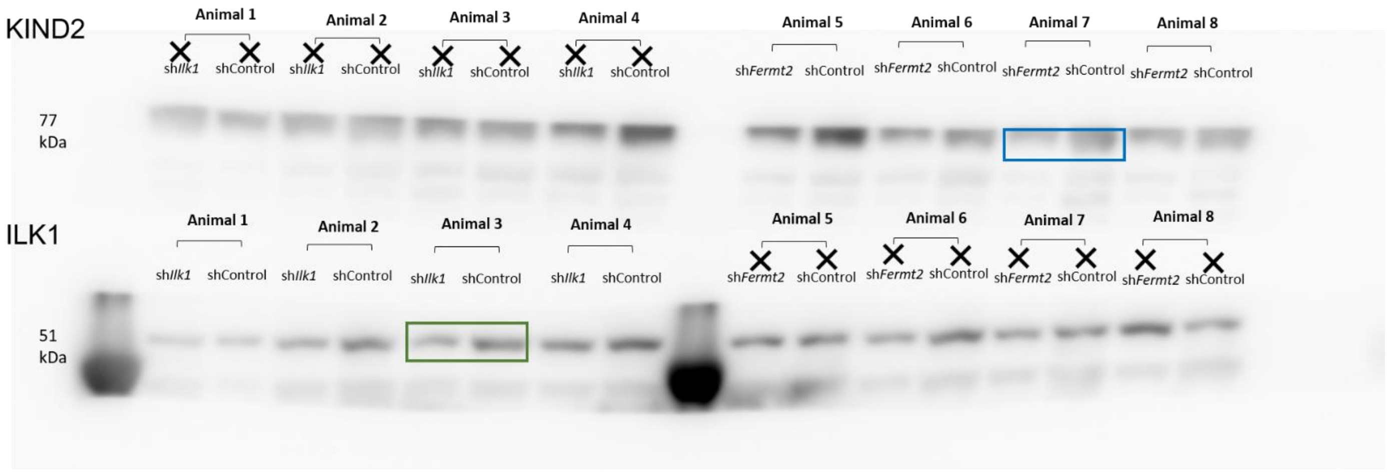


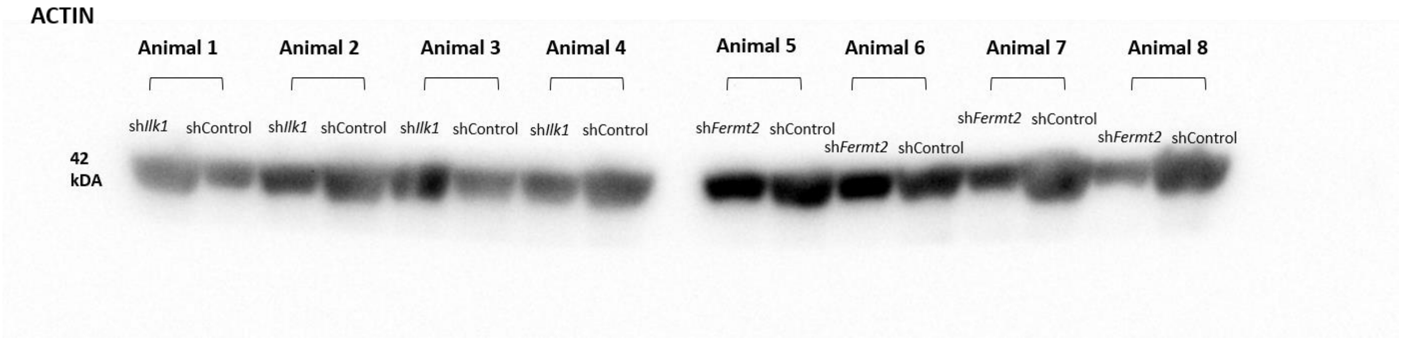

**FIGURE 3**

**Gel 1-5**: ACTIN

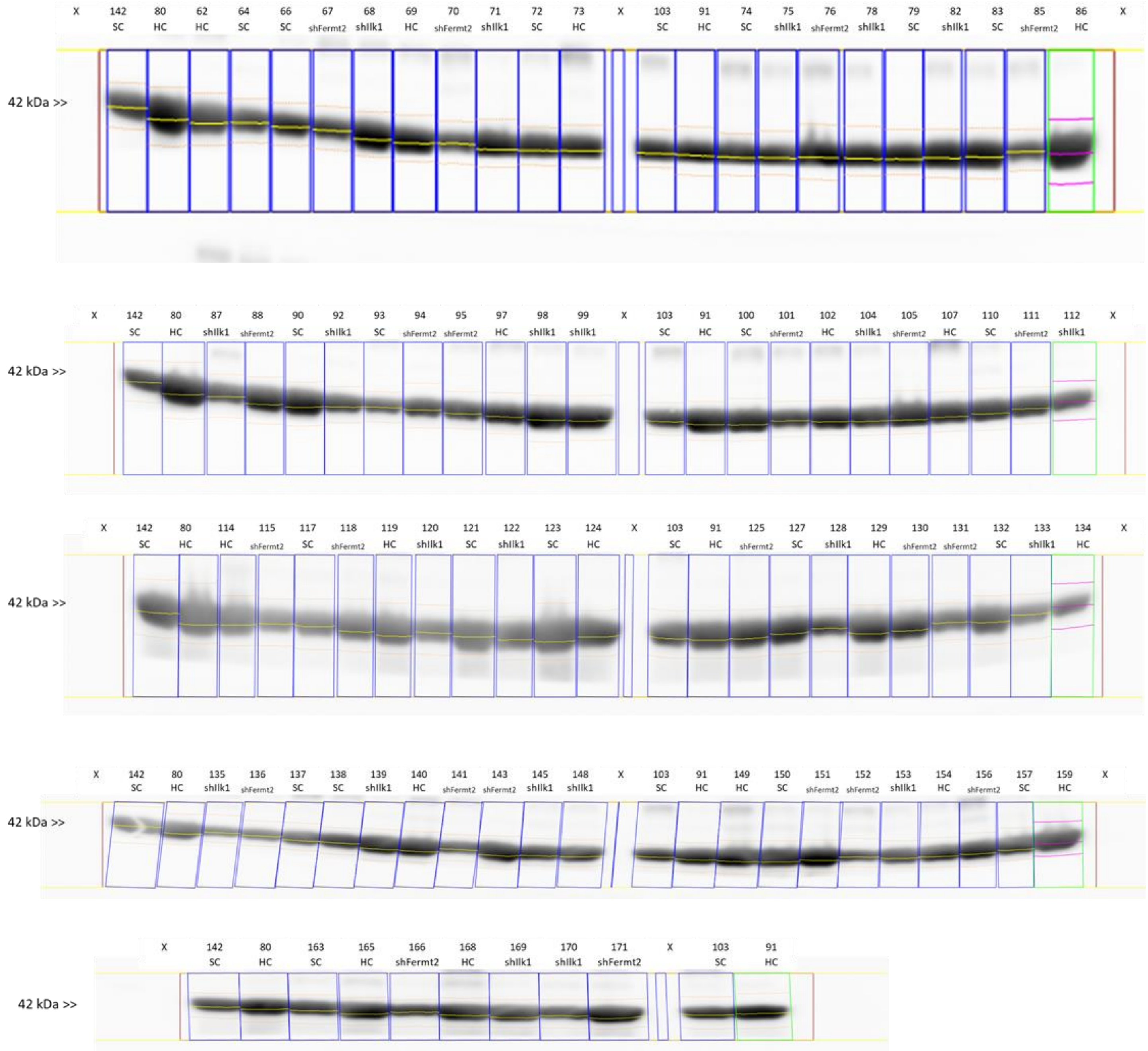


**Animal ID-number and randomization:**

- **HC**: Healthy shControl
- **SC**: Sepsis shControl
- **shIlk1**: Sepsis sh*Ilk1*
- **shFermt2**: Sepsis sh*Fermt2*

**Gel 1-5**: ILK1

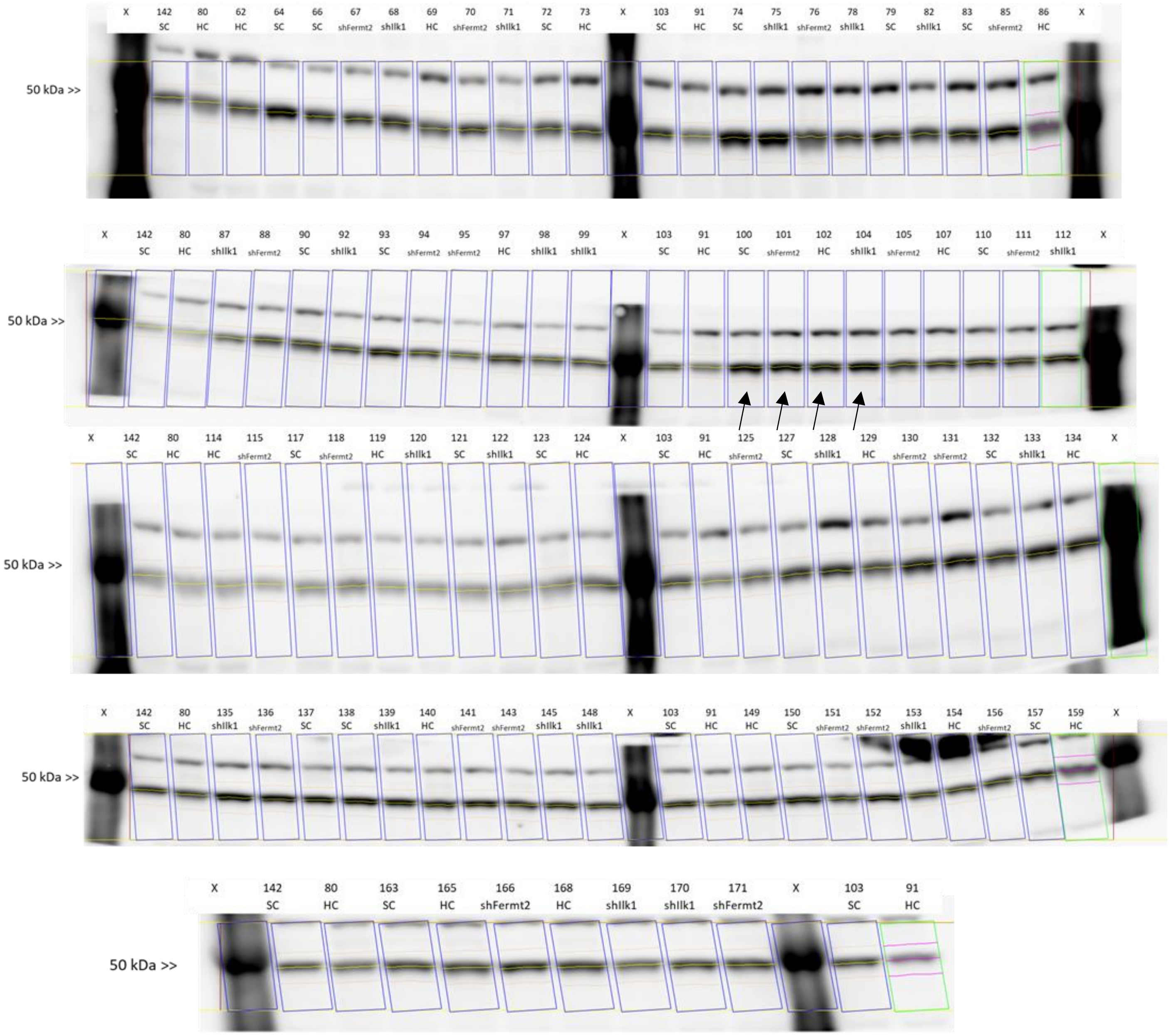


**Animal ID-number and randomization:**

- **HC**: Healthy shControl
- **SC**: Sepsis shControl
- **shIlk1**: Sepsis sh*Ilk1*
- **shFermt2**: Sepsis sh*Fermt2*

**Gel 1-5**: KINDLIN2

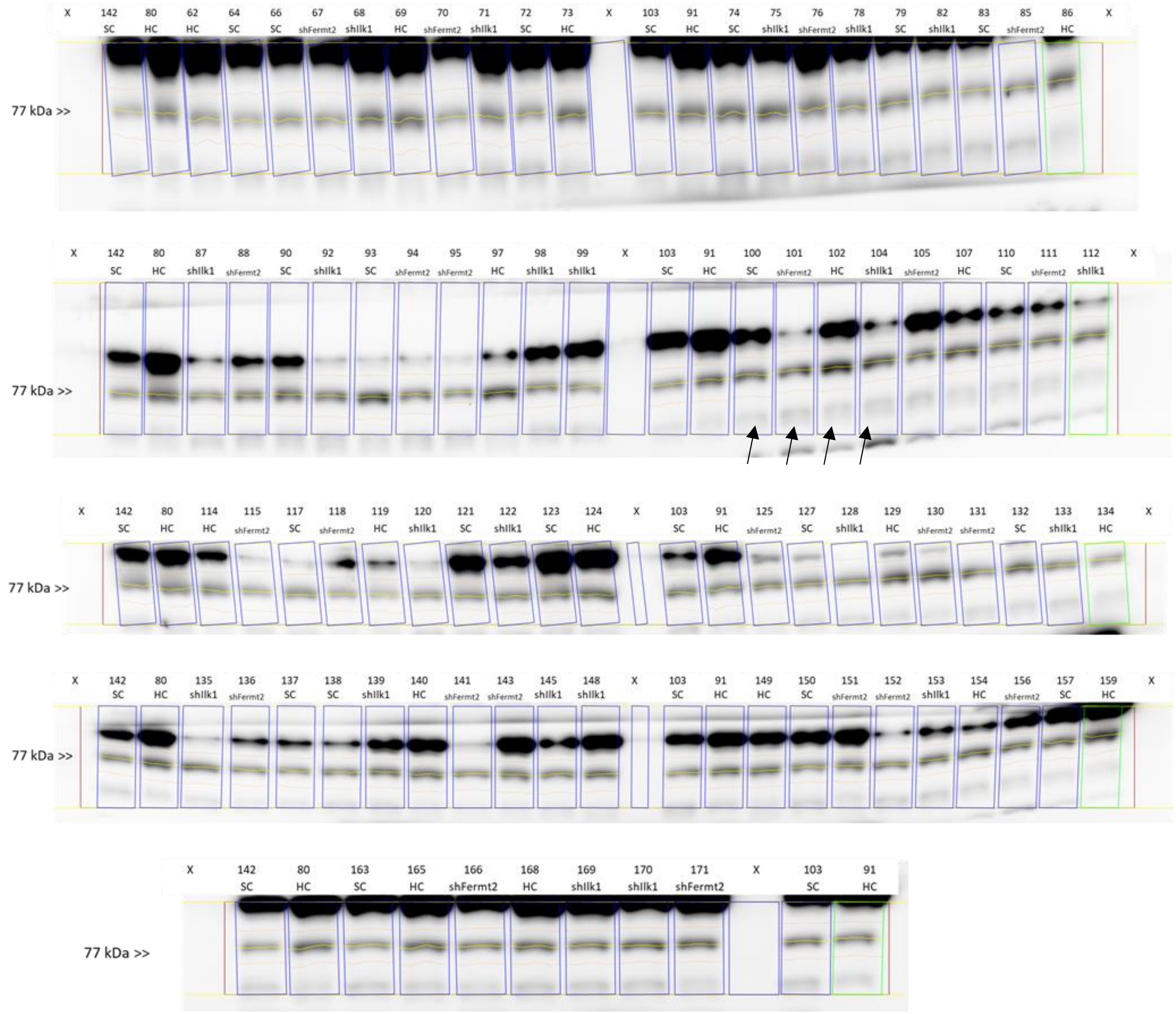


**Animal ID-number and randomization:**

**HC**: Healthy shControl
**SC**: Sepsis shControl
**shIlk1**: Sepsis sh*Ilk1*
**shFermt2**: Sepsis sh*Fermt2*
